# LMCD1 promotes osteogenic differentiation of human bone marrow stem cells by regulating BMP signaling

**DOI:** 10.1038/s41419-019-1876-7

**Published:** 2019-09-09

**Authors:** Bin Zhu, Feng Xue, Changqing Zhang, Guangyi Li

**Affiliations:** Department of Orthopaedics, Shanghai Jiao Tong University Affiliated Sixth People’s Hospital, NO.600 Yishan Road, 200233 Shanghai, China

**Keywords:** Mesenchymal stem cells, Stem-cell differentiation

## Abstract

Human bone marrow stem cells (BMSCs) are heterogeneous progenitor cells with two defining features, self-renew and multi-lineage differentiation. As one of the differentiation directions, osteogenesis is vital for bone homeostasis. A growing body of evidences show that ubiquitin-dependent protein degradation plays an essential role in the osteogenic differentiation of BMSCs. In this study, we found that LMCD1 was upregulated during osteogenic differentiation process of BMSCs by analyzing GSE80614. In vitro and in vivo functional studies confirmed that LMCD1 was critical to the osteogenic commitment of BMSCs. Compared to those of the controls, downregulation of LMCD1 significantly restrained osteogenic differentiation and enhanced adipogenic differentiation, while upregulation of LMCD1 increased the osteogenic differentiation and suppressed adipogenic differentiation. Mechanically, we found that LMCD1 could protect RUNX2 and Smad1 protein from Smurf1-induced ubiquitination degradation thereby regulating BMP signaling. In conclusion, our findings suggest that LMCD1 is a novel regulator of osteogenic differentiation and may be a potential therapeutic target for bone metabolism related diseases.

## Introduction

The human skeleton undergoes uninterrupted remodeling to maintain bone homeostasis, which mainly relies on a coordinated balance between bone resorption by osteoclasts and bone formation by osteoblasts^1,2^. Human bone marrow stem cells (BMSCs) are heterogeneous progenitor cells with the features of self-renewal capacity and multiple differentiation potential including adipogenesis, chondrogenesis and osteogenesis^3–6^. The osteogenic process of BMSCs is a critical step for bone formation. The process takes turns successively from osteoprogenitor cells to pre-osteoblasts, and eventually differentiate into mature osteoblasts^7,8^. Imbalanced bone homeostasis occurs if the process is disrupted. There seems to be an inverse relationship between osteogenesis and adipogenesis of BMSCs. Bone growth is enhanced when adipogenesis is inhibited in bone^9,10^. Thus, it is important to figure out how the BMSC differentiation process is regulated.

Bone morphogenetic proteins (BMPs) have been verified to play a critical role in osteogenic differentiation of BMSCs by several studies^11–13^. The activity of BMPs is realized through several intracellular signaling proteins and cell membrane receptors^7^. Among these, BMP/Smad signaling is one of the most pivotal pathways during this process. The binding reaction of BMPs and the type I BMP receptors activates and phosphorylates a group of transcription factors called receptor-regulated Smad (R-Smad) proteins, including Smad1, 5, and 8. The phosphorylated R-Smads then bind with Smad4, also known as common mediator Smad (co-Smad), and translocate into nucleus to activate the downstream transcription factors, such as Osterix (SP7) and Runt-related gene 2 (Runx2)^14–17^.

Protein ubiquitination system is an enzymatic cascade through which proteins are targeted for proteasomal degradation^18,19^. E1 (ubiquitin-activating enzymes), E2 (ubiquitin-conjugation enzymes) and E3 (ubiquitin ligases) are activated in sequence and precisely cooperate to modify protein activity through the process^20,21^. The ubiquitination system also plays an important role in mediating the osteogenic differentiation process of BMSCs. Smad ubiquitination regulatory factor 1 (Smurf1) can bind to Smad1, 5 and RUNX2, and induce their ubiquitination^22–24^. Therefore, Smurf1 is one of the most important negative regulators of BMP pathway and the osteogenic differentiation process of BMSCs.

LIM and cysteine-rich domains-1 (LMCD1) is a member of the LIM protein family, which contains an N-terminal cysteine-rich region, two C-terminal LIM domains and a central PET (Prickle, Espinas, and Testin) domain^25,26^. LMCD1 has been reported in cardiac tissues and lung acting as a transcriptional repressor for GATA6^27,28^. The mutations of LMCD1 promote cell migration and tumor metastasis in hepatocellular carcinoma^29^. In this study, we found that the expression of LMCD1 in BMSCs is upregulated during the osteogenic differentiation process. Further, in vitro and in vivo studies confirmed that BMSC osteoblast differentiation is regulated by LMCD1. Mechanically, we demonstrated that LMCD1 cooperates with Smurf1 to regulate the BMP/Smad signaling pathway.

## Results

### LMCD1 expression is upregulated during the osteogenic differentiation process of BMSCs

**Fig. 1:**
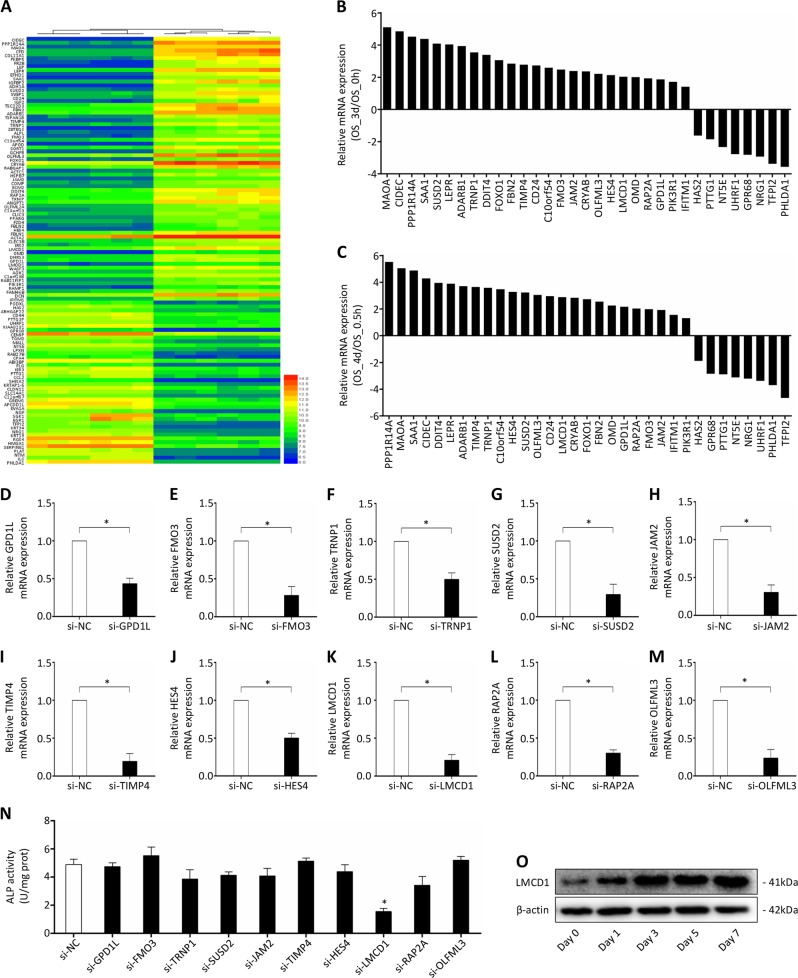
LMCD1 expression is upregulated during the osteogenic differentiation process of BMSCs. **a** Heat map of the regulated genes by comparing the expression at the differentiation time of 3 or 4 days with that at 0 or 0.5 h. **b**, **c** 33 genes were chosen to validate the reliability of the dataset by qRT-PCR. **d**–**n** 10 upregulated genes were chosen for functional screen by siRNA knockdown and ALP assay. **o** The expression of LMCD1 during the osteogenic differentiation process of BMSCs was measured by western blotting

To study the gene expression profile at different time phases during the BMSCs osteogenic differentiation process, we analyzed the dataset GSE80614 in this study. Totally 68 upregulated and 42 downregulated genes were identified by comparing the gene expression at the differentiation time of 3 or 4 days with that at 0 or 0.5 h (Fig. 1a). To further validate the reliability of the dataset, we chose 25 upregulated and 8 downregulated genes for qRT-PCR analysis. The results showed that the mRNA expression levels of these genes were consistent with the dataset (Fig. 1b, c). Among the 33 genes, MAOA, SAA1, ADARB1, FBN2, OMD, OGR1, NRG, UHRF1, CIDEC, DDIT4, LEPR, FOXO1, IFITM1, and HAS2 have been reported to be involved in osteogenic differentiation, osteoporosis and bone homeostasis^30–42^, which further validated the reliability of this dataset. Then we chose ten of the other upregulated genes for functional screen by siRNA knockdown and ALP activity assay (Fig. 1d–m). We noticed that knocking down of LMCD1 expression in BMSCs significantly reduced the ALP activity (Fig. 1n). Besides, the protein expression level of LMCD1 was upregulated during the osteogenic differentiation process of BMSCs (Fig. 1o). In sum, we hypothesized that LMCD1 may be associated with the osteogenic differentiation of human BMSCs.

### The function of LMCD1 in osteogenic differentiation in vitro

**Fig. 2:**
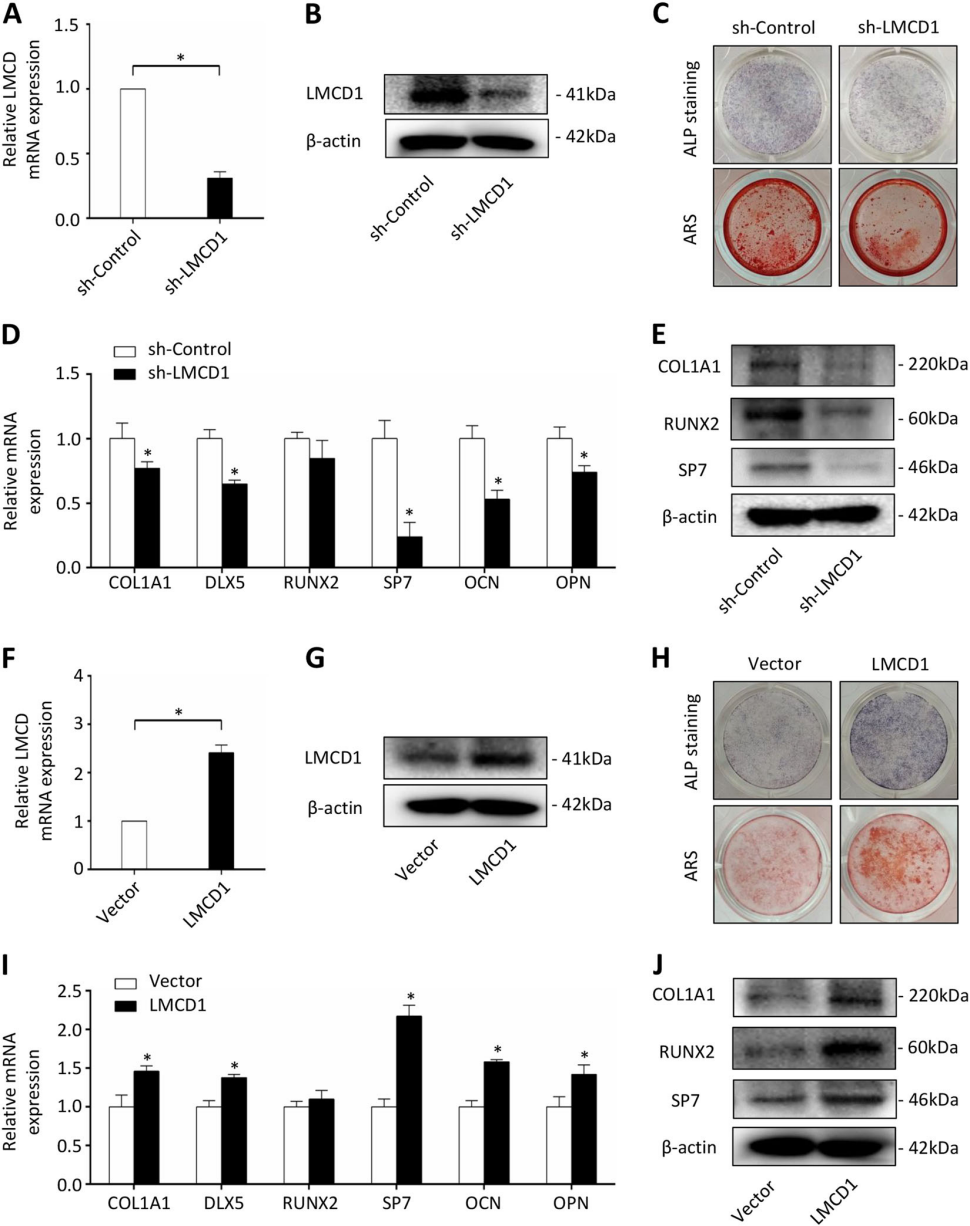
The function of LMCD1 in osteogenic differentiation in vitro. **a**, **b** The efficacy of LMCD1 specific shRNA was measured by qRT-PCR and western blotting. **c** ALP staining and Alizarin red staining were performed after treating BMSCs with osteogenic induction media for 7 days or for 14 days. **d**, **e** The expression of osteogenic markers was assessed by qRT-PCR and western blotting. **f**, **g** The efficacy of LMCD1 specific lentivirus was measured by qRT-PCR and western blotting. **h** ALP staining and Alizarin red staining were performed after treating BMSCs with osteogenic induction media for 7 days or for 14 days. **i**, **j** The expression of osteogenic markers was assessed by qRT-PCR and western blotting

To examine whether LMCD1 played important roles in osteogenic differentiation of BMSCs, LMCD1 specific shRNA was used to knockdown its expression. Western blotting and qRT-PCR were used to determine the efficacy (Fig. 2a, b). Next, ALP staining and Alizarin red staining were performed after treating BMSCs with osteogenic induction media for 7 days or for 14 days. We found that both the ALP activity and the mineralized nodule formation of the differentiating BMSCs were inhibited by knocking down LMCD1 expression compared to those of controls (Fig. 2c). The expression of several osteogenic markers was also assessed at mRNA and protein levels after 7 days of osteogenic media treatment. Knockdown of LMCD1 significantly inhibited the mRNA expression of COL1A1, DLX5, SP7, OCN, OPN (Fig. 2d) and reduced the protein expression of COL1A1, RUNX2, SP7 (Fig. 2e). To further examine the function of LMCD1 in osteogenic differentiation of BMSCs, we upregulated its expression using LMCD1 specific lentivirus. The efficacy of the infection was determined by western blotting and qRT-PCR (Fig. 2f, g). Then, ALP staining and Alizarin red staining were performed, and we found that high expression of LMCD1 significantly improved the ALP activity and mineralized nodule formation of BMSCs (Fig. 2h). Besides, the mRNA expression of COL1A1, DLX5, SP7, OCN, OPN (Fig. 2i) and the protein expression of COL1A1, RUNX2, SP7 were increased in response to the upregulating the LMCD1 expression (Fig. 2j).

### The role of LMCD1 in osteogenic differentiation in vivo

**Fig. 3:**
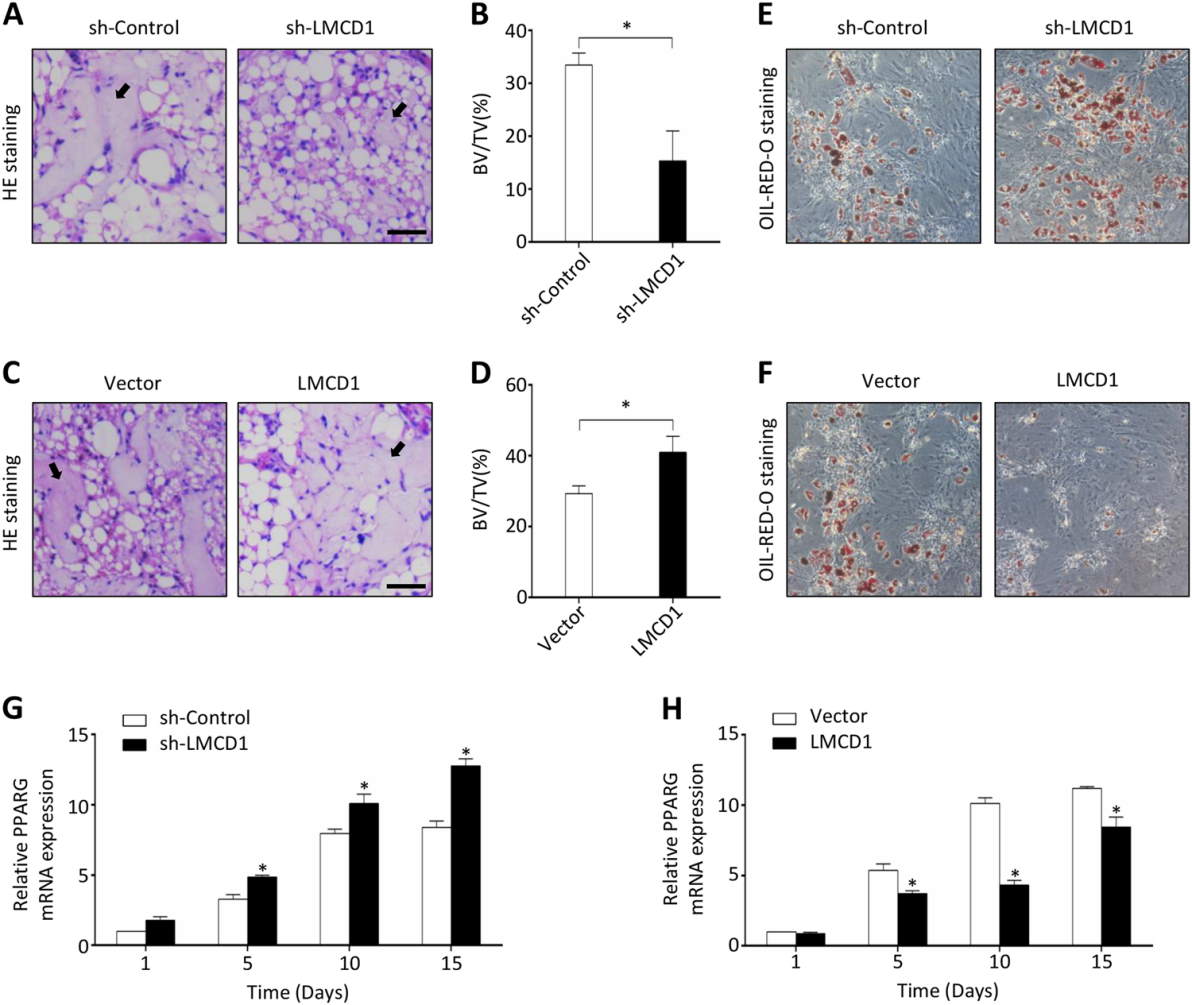
The role of LMCD1 in osteogenic differentiation in vivo. **a** Representative H&E staining images of transplants from sh-Control group and sh-LMCD1 group. Black arrow represents newly formed bone. Scale bar represents 100 µm. **b** Quantitative measurement of bone formation. **c** Representative H&E staining images of transplants from Vector group and LMCD1 group. Black arrow represents newly formed bone. Scale bar represents 100 µm. **d** Quantitative measurement of bone formation. **e**, **f** The adipogenic differentiation was measured by Oil-red-O staining after BMSCs were cultured with adipogenic induction medium for 14 days. **g**, **h** The expression of PPARG was measured by qRT-PCR during the adipogenic differentiation process of BMSCs

To verify the findings of our in vitro experiments, we further examined the effects of LMCD1 on bone formation in vivo. BMSCs with stably upregulated or downregulated LMCD1 expression were mixed with β-TCP (tricalcium phosphate) and then transplanted into muscle pockets on the hind limbs of 8-week-old immunocompromised mice. The transplants were harvested for histological analysis eight weeks later. We found that BMSCs with downregulated LMCD1 expression formed less bone tissues than those in the corresponding control group (Fig. 3a), while BMSCs with upregulated LMCD1 expression formed more bone tissues than those in their corresponding control group (Fig. 3c). The quantitative measurement of bone tissue area revealed the differences between groups with statistical significance (Fig. 3b, d). These results were further confirmed by Masson staining and OCN immunofluorescence (Fig. S1 A-H). In addition, we also noticed that the adipose tissue formation was increased in the LMCD1-downregulated group while decreased in the LMCD1-upregulated group when compared with those in their corresponding control groups in some regions (Fig. 3a, c), which hint us that LMCD1 may have an inhibition effect of adipogenic differentiation of BMSCs.

The osteogenic and adipogenic differentiation of BMSCs are in a dynamic balance and contribute to bone homeostasis. To further confirm the effect of LMCD1 on BMSC adipogenic differentiation, we performed the Oil-red-O staining after BMSCs were cultured with adipogenic induction medium. We found that downregulation of LMCD1 expression significantly enhanced the adipogenesis effect (Fig. 3e), while upregulation of LMCD1 expression significantly reduced the adipogenesis effect (Fig. 3f). Besides, we also assessed the mRNA expression of PPARG, a master adipogenic transcription factor for adipogenesis. As shown in Fig. 3g, h, the expression of PPARG was significantly enhanced by the downregulation of LMCD1 expression and suppressed by the upregulation of LMCD1 expression. The PPARG immunofluorescence further confirmed the conclusion (Fig. S1 I-L).

### LMCD1 regulates osteogenic differentiation of BMSCs via the BMP signaling

**Fig. 4:**
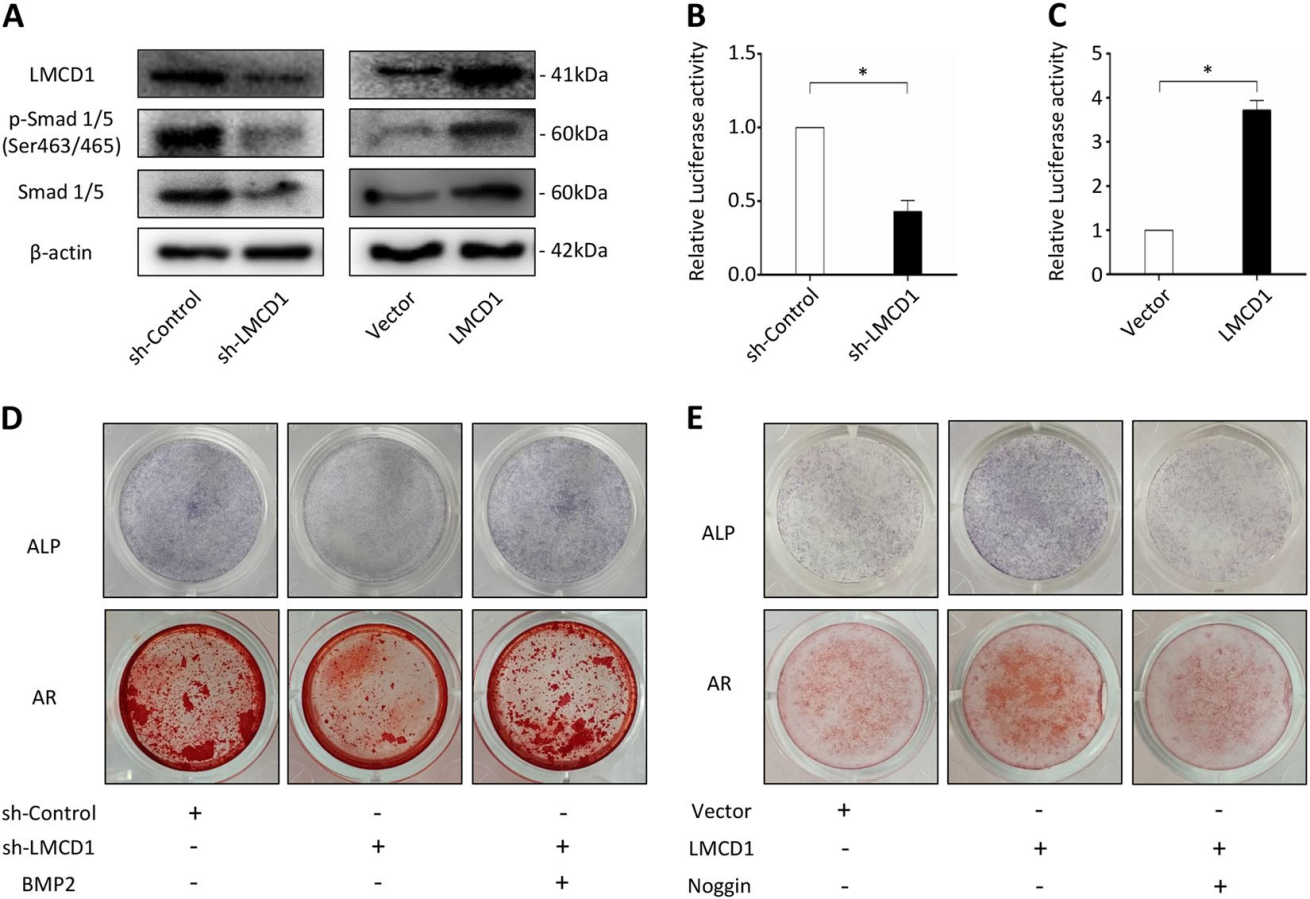
LMCD1 regulates osteogenic differentiation of BMSCs via the BMP signaling. **a** The expression of Smad1, 5, and their phosphorylation levels were measured with the upregulation or downregulation of LMCD1. **b**, **c** Relative BRE Luciferase activity was measured with the upregulation or downregulation of LMCD1 in 293T cells. **d** ALP staining and AR staining showed that the restricted osteogenic capacity of LMCD1-downregulated BMSCs was rescued by BMP2 treatment. **e** ALP staining and AR staining showed that the enhanced osteogenic ability of LMCD1-upregulated BMSCs was reduced by Noggin treatment

BMP signaling plays a vital role in osteogenic differentiation of BMSCs and bone homeostasis. As we previously demonstrated that the well-known target genes of BMP signaling, including RUNX2, SP7, and DLX5, were significantly influenced by the expression of LMCD1, we hypothesized that LMCD1 regulated osteogenic differentiation of BMSCs through the BMP signaling. To this end, we examined the expression of Smad1, 5, and their phosphorylation levels. We found that both the expression of Smad1, 5, and their phosphorylation levels were decreased with the downregulation of LMCD1 expression in BMSCs, but were increased with the upregulation LMCD1 expression (Fig. 4a). Then we performed a BRE luciferase assay, which contains sensitive BMP-responsive elements^43^. We found that the downregulation of LMCD1 expression in 293T cells significantly reduced the BMP2-induced response, while the upregulation of LMCD1 expression enhanced it (Fig. 4b, c). Besides, ALP staining and AR staining showed that the restricted osteogenic capacity of LMCD1-downregulated BMSCs was rescued by BMP2 treatment, while the enhanced osteogenic ability of LMCD1-upregulated BMSCs was reduced by Noggin treatment, an antagonist of bone morphogenetic proteins^44,45^ (Fig. 4d, e).

### LMCD1 is essential for RUNX2 and Smad1 stabilization

**Fig. 5:**
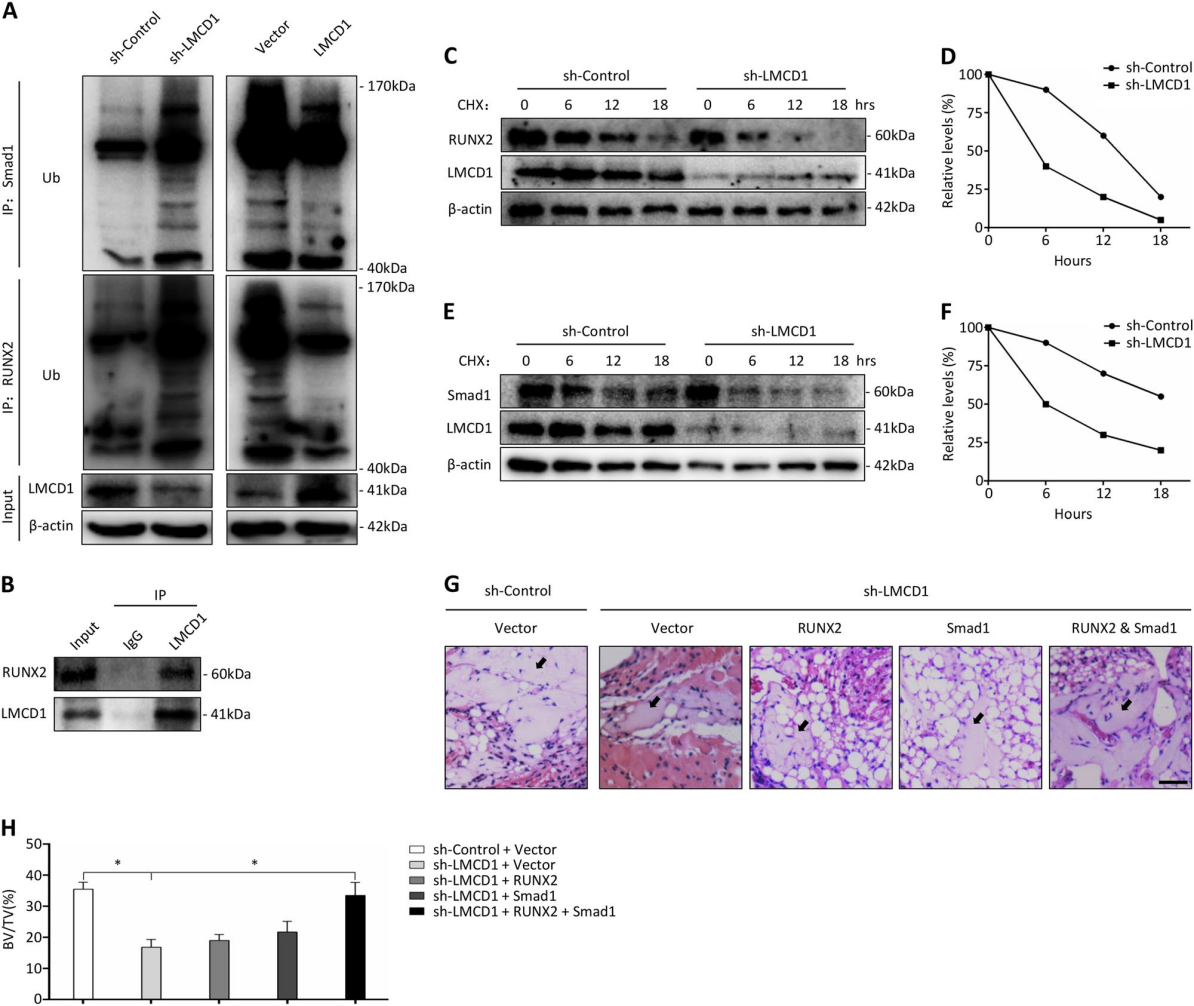
LMCD1 is essential for RUNX2 and Smad1 stabilization. **a** RUNX2-linked and Smad1-linked ubiquitin was measured by immunoblotting in BMSCs with upregulation or downregulation of LMCD1 expression. **b** The interaction of LMCD1 and RUNX2 in BMSCs was detected by co-immunoprecipitation. **c**, **d** The expression of RUNX2 was measured with the presence of cycloheximide (CHX) at different time points by western blotting. **e**, **f** The expression of Smad1 was measured with the presence of CHX at different time points by western blotting. **g** Representative H&E staining images of transplants from different groups. Black arrow represents newly formed bone. Scale bar represents 100 µm. **h** Quantitative measurement of bone formation

The ubiquitin-proteasome system is the main pathway of protein post-translational modification and plays important roles in controlling the stability and activity of many proteins. As we have shown that the RUNX2 protein expression was significantly regulated by the expression of LMCD1, while its mRNA expression barely changed (Fig. 2d, e, Fig. 2i, j), we hypothesized that LMCD1 may influence the ubiquitination degradation of RUNX2. To determine the underlying mechanism, we performed the co-immunoprecipitation assay and found that LMCD1 could physically interact with RUNX2 (Fig. 5b). Then we examined the ubiquitination of RUNX2 in MG132-treated BMSCs and found that the downregulation of LMCD1 enhanced the ubiquitination level of RUNX2 while the upregulation of LMCD1 reduced it (Fig. 5a). Besides, we also examined the stability of RUNX2 protein in the presence of cycloheximide (CHX), a protein synthesis inhibitor. We found that the half-life of RUNX2 protein was significantly reduced in LMCD1-knockdown cells in comparison with that of the control group (Fig. 5c, d). In addition, we also discovered that LMCD1 could reduce the ubiquitination degradation of Smad1, a key protein in BMP signaling for inducing the downstream transcription factors such as RUNX2, SP7, and DLX5 (Fig. 5a) and shorten the half-life of Smad1 protein (Fig. 5e, f).

Then we transduced LMCD1-downregulated BMSCs with RUNX2-lentivirus, Smad1-lentivirus or a combination of both lentiviruses to overexpress RUNX2 and Smad1, and we transplanted them into muscle pockets on the hind limbs of 8-week-old immunocompromised mice. The histological analysis confirmed that only the combined overexpression of RUNX2 and Smad1 could restore the bone formation capacity of LMCD1-downregulated BMSCs (Fig. 5g, h).

### LMCD1 cooperates with Smurf1 to regulate the BMP signaling

**Fig. 6:**
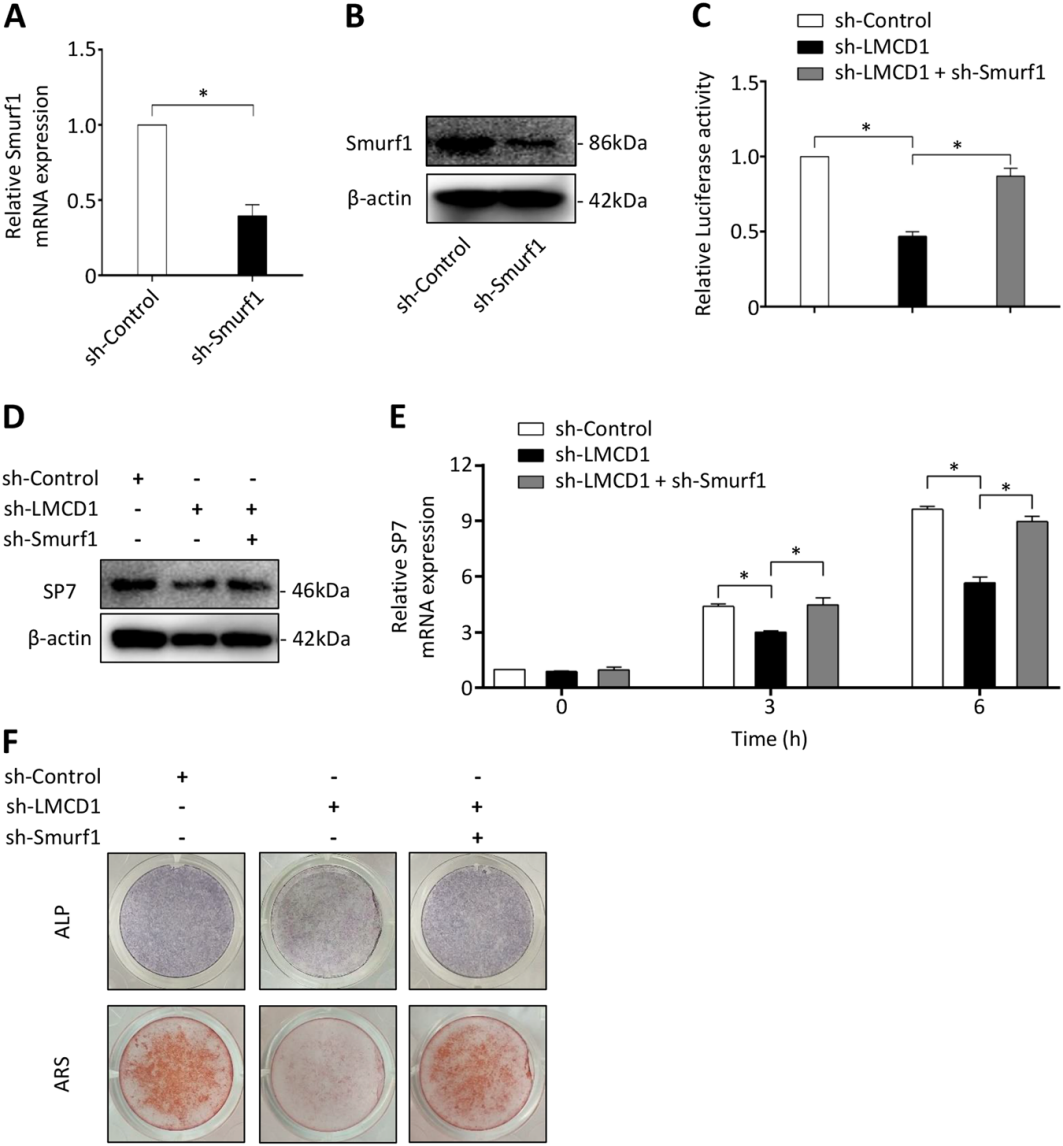
LMCD1 cooperates with Smurf1 to regulate the BMP signaling. **a**, **b** The efficacy of Smurf1 specific shRNA was measured by qRT-PCR and western blotting. **c** Relative BRE Luciferase activity was measured in 293T cells. **d**, **e** The expression of SP7 was measured by western blotting and qRT-PCR with the depletion of LMCD1 or depletion of both LMCD1 and Smurf1. **f** ALP staining and Alizarin red staining were performed with the depletion of LMCD1 or depletion of both LMCD1 and Smurf1

Smurf1 is an E3 ubiquitin ligase. It induces RUNX2 and Smad1 degradation by directly interacting with these proteins, and eventually restrains the BMP signaling^46,47^. We hypothesized that Smurf1 and LMCD1 might cooperate to regulate the BMP signaling. So, we downregulated the Smurf1 expression using a Smurf1 specific shRNA in cells, and the efficacy was confirmed by qRT-PCR and western blotting assays (Fig. 6a, b). We found that downregulation of Smurf1 expression significantly promoted the BMP2-inducted BRE luciferase activity in 293T, and upregulated the expression of RUNX2, p-Smad1/5 (Ser463/465) (Fig. S2 A, B). Besides, knockdown of Smurf1 expression successfully rescued the BMP2-induced luciferase activity in LMCD1-downregulated cells compared to that of controls (Fig. 6c). The expression of SP7 at mRNA and protein levels was measured in consideration of its major role in BMP signaling, and we observed that the reduction of SP7 expression in LMCD1-downregulated BMSCs was regained by sh-Smurf1 (Fig. 6d, e). Similarly, the reduction of RUNX2 and p-Smad1/5 (Ser463/465) expression in LMCD1-downregulated BMSCs was also regained by sh-Smurf1 (Fig. S2C). In addition, the BMP2-induced ALP activity and calcium mineralization were restored in LMCD1-downregulated BMSCs by depletion of Smurf1 (Fig. 6f).

## Discussion

Human bone marrow stem cells have multiple differentiation capabilities, and they can differentiate into multiple tissues under certain conditions including bone, fat and cartilage^6,48–50^. Increasing evidence has affirmed that the osteogenic differentiation of BMSCs, especially the inverse relationship between osteogenesis and adipogenesis of BMSCs is essential for bone homeostasis^51–53^. It is urgent to figure out the underlying molecular mechanisms that regulate their differentiation in order to explore novel therapeutic targets for bone metabolism related diseases.

In this study, we analyzed the dataset GSE80614, which consists data of gene expression profiles in the early stage during human bone marrow stem cell osteogenic differentiation^54^. Sixty-eight upregulated and forty-two downregulated genes were identified when we compared the expression profile during BMSC osteogenic differentiation after 3 or 4 days versus that at 0 or 0.5 h. Several identified genes have been reported to be closely related to osteogenesis and bone homeostasis, which further validated the reliability of this dataset. Twenty-five upregulated and eight downregulated genes were chosen to verify the micro-array results, and ten of them were selected for functional screen by siRNA transfection and ALP activity assay. The results showed that knockdown of LMCD1 significantly reduced ALP activity after the osteogenic differentiation of BMSCs.

Previous studies have confirmed the function of LMCD1 in cardiac hypertrophy, thrombin formation, and hepatocellular carcinoma migration^29,55,56^. However, the role of LMCD1 in osteogenic differentiation of BMSCs and bone homeostasis has never been reported before. In this study, we showed that the osteogenic differentiation potency of BMSCs, as measured by ALP staining and AR staining, was closely related to the expression of LMCD1 in vitro. The BMSC transplant assay with immunocompromised mice also confirmed these results in vivo. Besides, we noticed that the expression of LMCD1 exhibited an inverse correlation with the adipogenic differentiation capacity of BMSCs. These data indicate that LMCD1 may play a pivotal role in osteoblastic differentiation of BMSCs and in skeletal homeostasis.

The BMP signaling pathway, which is indispensable for osteogenesis, was regulated by downregulation or upregulation of LMCD1 expression. Smad 1/5/8, as well as the subsequent transcription factors, including Runx2 and Sp7, are the main downstream elements through which BMPs triggers the osteogenic differentiation of BMSCs^57,58^. We found that LMCD1 could interact with RUNX2 and Smad1, and reduce their ubiquitination degradation. Smurf1 is an E3 ligase that mediates Smad1 and RUNX2 degradation^59–61^. In addition, Smurf1 is the first E3 ligase that has been verified to be involved in RUNX2 ubiquitination. Zhao et al.^62^ found that the osteoblast activity was enhanced in Smurf1 deficient mice and the bone mass was also increased. The results were also confirmed by Yamashita and his colleagues^22^. In this study, we found that knockdown of Smurf1 expression is capable of rebuild the osteogenic capacity of LMCD1-deficient BMSCs, which give us a hint that LMCD1 cooperates with Smurf1 to regulate the BMP signaling during the osteoblast differentiation process.

Collectively, we found a positive relationship between the osteogenic ability of BMSCs and the expression of LMCD1 in vitro and in vivo, which regulates the BMP signaling during osteogenic differentiation process. Mechanically, LMCD1 cooperates with Smurf1 to modulate the ubiquitination levels of Smad1 and RUNX2. These data demonstrate that LMCD1 is a novel regulator of osteogenic differentiation and may be a potential therapeutic target for bone metabolism related diseases.

## Materials and methods

### Microarray analysis

The dataset GSE80614, which consists data of gene expression profiles in the early stage of human bone marrow stem cell osteogenic differentiation, was obtained from the GEO database (https://www.ncbi.nlm.nih.gov/geo/). The expression profile at different time point (3 and 4 days versus 0 and 0.5 h) were analyzed with *p*-value < 0.01 and |logFC| >2 set as the threshold.

### Cell culture

Human bone marrow stem cells (BMSCs) were obtained from a healthy donor (aged 43 years) who underwent amputation for severe trauma, and were cultured in a-MEM (Sigma-Aldrich, Missouri, USA). The 293T cell line was obtained from the American Tissue Culture Collection (ATCC) and was cultured in DMEM (Sigma-Aldrich, Missouri, USA). Each type of medium contained 10% fetal bovine serum (FBS) (Gibco, California, USA) and 100 µg/ml penicillin-streptomycin sulfate (Sigma-Aldrich, Missouri, USA). Cells were maintained at 37 °C with 5% carbon dioxide.

### In vitro differentiation assays

Osteogenic differentiation medium (Cyagen, Guangzhou, China) and adipogenic differentiation medium (Cyagen, Guangzhou, China) were used in accordance with the operating manual to induce BMSC differentiation. Briefly, cells were cultured in 24-well plates with osteogenic differentiation medium or adipogenic differentiation medium. The medium was replaced every other day. Osteogenic differentiation was evaluated with quantitative Real Time PCR (qRT-PCR), western blot, Alkaline phosphatase (ALP) activity assay, ALP staining, and Alizarin red (AR) staining. Adipogenic differentiation was evaluated with qRT-PCR, western blot and Oil Red O staining.

### RNA isolation and qRT-PCR assays

Total RNA extraction was performed with the aid of TRIzol Reagent (Invitrogen, Carlsbad, USA) according to the operating instructions. Total RNA was quantified by NanoDrop 2000 (Thermo, Waltham, USA) and reverse-transcribed into cDNA using PrimeScriptRT Reagent kit (TaKaRa, Shiga, Japan). Gene expression levels were determined by qTR-PCR using ABI HT7900 (Applied Biosystems, Australia). The expression of β-actin was used for normalization. The sequences of the primers are listed in Table S1.

### Western blot analysis and Co-immunoprecipitation

Protein lysates were extracted from BMSCs with Cell Lysis Buffer containing Protease Inhibitor (Boster, Wuhan, China) and quantified using a BCA Protein Assay Kit (Thermo, Waltham, USA). Proteins were separated with electrophoresis at 130 V for 70 min on a 12% SDS-PAGE gel (sodium dodecyl sulfate polyacrylamide gel electrophoresis, EpiZyme, Cambridge, MA), and transferred to a PVDF membrane (polyvinylidene difluoride membrane, Millipore, MA, USA) by electroblotting at 280 mA for 75 min. The membranes were blocked for 70 min in 5% nonfat milk. Primary antibodies incubation was performed at 4 °C for overnight, and secondary antibodies incubation was performed at room temperature for 1 h. Protein expression were detected using Image Quant LAS 4000 (GE Healthcare). Antibodies are listed in table S2.

For co-immunoprecipitation, we used an immunoprecipitation kit (Abcam, Cambridge, MA, USA). Briefly, cells were lysed in Lysis Buffer supplemented with Protease Inhibitor Cocktail on ice for 20 min and centrifuged for 10 min at 1000×*g*. The supernatant was gathered and incubated with antibodies overnight at 4 °C, and then incubated with Protein A/G Sepharose beads for 1 h at 4 °C. The beads were collected with low speed centrifugation and washed for 3 times with Wash Buffer. Co-precipitated proteins were eluted with SDS-loading buffer at 95 °C for 5 min, and analyzed with western blotting.

### Plasmid transfection and reporter gene activity assay

Specific siRNAs were transfected into cells using Lipofectamine 2000 Reagent (Invitrogen, California, USA) according to the operating protocol. Cells were ready for use 24 h after transfection. For virus productions, 12 µg plasmids for LMCD1, RUNX2, Smad1, sh-LMCD1 and corresponding control, 9 µg packaging plasmid and 3.6 µg envelope plasmid were transfected into 293T cells using Lipofectamine 2000 Reagent. Viruses were collected 48 h after the transfection and filtered with a 0.45 µm filter (Sigma-Aldrich, Missouri, USA). BMSCs were infected in the presence of 6 µg/ml polybrene (Sigma-Aldrich, Missouri, USA). For the reporter gene activity assay, 293T cells were transfected with LMCD1 siRNA or control siRNA, 100 ng BRE luciferase and 50 ng β-galactosidase using Lipofectamine 2000 Reagent. Twenty-four hours later, cells were serum starved overnight and then treated with 100 ng/ml BMP2 for 6 h. Cells were lysed, and the luciferase activity was measured using a luciferase assay system (Promega, Wisconsin, USA).

### In vitro ubiquitination assay

Cells were treated with MG132, a proteasome inhibitor, for 8 h and then they were collected using Cell Lysis Buffer. Antibodies were added to the lysate and incubated at 4 °C for 3 h. Then the lysate was gently rotated at 4 °C for 10 h after protein A/G Sepharose beads were added. The beads were collected with low speed centrifugation and washed 3 times with Wash Buffer. The immunoprecipitated proteins were eluted with SDS-loading buffer at 95 °C for 5 min, and analyzed with western blotting.

### Animal studies

Eight-week-old immunocompromised mice were used in this experiment and all procedures were approved by the Ethics Committee of Shanghai Sixth People’s Hospital. BMSCs (5 × 10^6^) were seeded on beta-tricalcium phosphate (β-TCP, Bio-lu, Shanghai, China) and transplanted into muscle pockets on the hind limbs. Eight weeks later, animals were put to death by overdose anesthesia, and the transplants were harvested and fixed in 10% formalin. For histological analysis, the transplants were decalcified with 10% EDTA and embedded in paraffin. Samples were sliced into 5 µm sections using Leica RM2235 (Leica, Heidelberg, Germany) for Hematoxylin and Eosin staining. Images were collected and analyzed with BIOQUANT OSTEO (BIOQUANT, Nashville, TN, USA).

### Statistical analysis

All values are expressed as means ± SD (standard deviation). Three separate replicates were carried out to confirm all results. Statistical differences were evaluated using an independent-sample *t*-test or one-way analysis of variance (ANOVA) for comparison between two groups or between more than two groups, respectively. Statistical analysis was performed using SPSS 16.0 (IBM Corporation, New York, USA). (*p < 0.05).

## Supplementary information

Supplementary

LMCD1 figure legends for supplementary

Figure S1

Figure S2
