# Supplementary material for: LMCD1 promotes osteogenic differentiation of human bone marrow stem cells by regulating BMP signaling
Source: Cell Death Dis. 2019 Sep 9;10(9):647. doi: 10.1038/s41419-019-1876-7 (PMC6733937; doi:10.1038/s41419-019-1876-7)
Supplement: Supplementary file 1 — Supplementary [file 41419_2019_1876_MOESM1_ESM.docx]

Table 1. The sequences of the primers

| Gene Symbol | Forward primer | Reverse primer | Product length |
| --- | --- | --- | --- |
| MAOA | CCCGGAGTATCAGCAAAA | CAAAGGGTGGGAAGGAC | 127 |
| CIDEC | GAGCCAGGGGATGAGAAA | GGAGAGGGACTTGGGGTAG | 100 |
| SAA1 | CTATGATGCTGCCAAAAGG | CTCCTGCCCCATTCATT | 141 |
| PPP1R14A | AGCGGCCTACTCTCGCA | CCGGGTGCTTCTTACGG | 127 |
| SUSD2 | TACTACGGCACCGCCAA | AGGGCATTCCTGTCTCCTC | 115 |
| LEPR | AGATTCACCTCTGGTTCCC | GTCGTTGAGTTTGGCTGTT | 112 |
| ADARB1 | GGCCATTCATTCAGGTTTT | TCACCCACATCCATTCG | 128 |
| TRNP1 | TGTTCGGGTGTGCTGTG | GTCAAGGTCGGAGTTGGG | 196 |
| DDIT4 | CTCTTCGCCCTCGTCCT | CCACTGTTGCTGCTGTCC | 147 |
| FOXO1 | TTATGCGAACAGACCAACC | GGTAGCGAAATGCAGGAG | 128 |
| FBN2 | AACCACAGGTGCCAACA | CAGGAAGCAGAGCCACA | 146 |
| TIMP4 | CTCCCAAACCCCATTAGTC | CGCCATTTCTCCCCTAC | 150 |
| CD24 | CCAGCCATCAAAATGCTT | TTCCAGTCTTCACTTCCCA | 163 |
| C10orf54 | CCTGGGCATTTGTCTCCT | GGTGCTCCCCTTTTCCA | 138 |
| FMO3 | TTATCCTTGGGACATGCTG | GCATTCATCTGCTTCACG | 102 |
| JAM2 | AAATGCCTAATGGCTGGA | CTTGGGTTTGTGTTTGACC | 107 |
| CRYAB | GAGTCCCTTCTACCTTCGG | CCATGCACCTCAATCACA | 174 |
| OLFML3 | TCTGGCACCACTCTCCA | GTCGGCGTTCCATGTACT | 124 |
| HES4 | TGGACGCCCTCAGAAAAGA | CGCGGTACTTGCCCAGA | 150 |
| LMCD1 | TCCTCCATCTCCAAGCC | GGCCTTTTATGCGTCACT | 137 |
| OMD | GGGAAAAGAAAGAAGTAAGCC | GAAAACACTCGGGTTGAATTA | 120 |
| RAP2A | AATGGTGGACGAACTCTTTG | CAGAACAGCATGGGTCATC | 83 |
| GPD1L | GTCATTCCCCACCAGTTC | CCCTCGTCTATGCCCTT | 101 |
| PI3KR1 | CCCGCCTCTTCTTATCAA | CGATCATTTCCAAGTCCAC | 149 |
| IFITM1 | CACGCAGAAAACCACACTT | CAGCACAGCCACCTCAT | 93 |
| HAS2 | TGAGGACGACTTTATGACCA | TGATTCCAAGGAGGAGAGAG | 112 |
| PTTG2 | GCCAAGGATGTGCTGAA | TTGACAGTGCCCAAAGC | 146 |
| NT5E | AGGAAGGGGAAGAACAGG | GCTAATGCCGTGTGTCAG | 120 |
| UHRF1 | CAGAAATGGCCTCAAGGG | AAGGAACGAATCAAAGGCA | 128 |
| GPR68 | GCTTGAGGGTGGTGGTTC | CTGGTGGTGGGCAAGAC | 135 |
| NRG1 | TGAATGGAGGGGAGTGCT | CAGGCAGAGACAGAAAGGG | 150 |
| TFPI2 | TTCTTGGGGTCGTATTCCT | TGTTGTTAATCTGCGACTTTG | 179 |
| PHLDA1 | CAAGAGGACGGCAGAGG | GAGGCTAACACGCAGGAG | 123 |
| COL1A1 | CCTGCTGGCAAGAGTGGT | GCCCTGTTCGCCTGTCT | 135 |
| DLX5 | GCTATGCACCATCCGTCT | GGGTTGAGAGCTTTGCC | 140 |
| RUNX2 | CCCTTTTGCTGCTGTCTC | CTGGAAGAATGCCACACAC | 127 |
| SP7 | CCCCATCTCCCTTGACTG | TTCCCCAAAGAGCACATCT | 136 |
| OCN | CACACTCCTCGCCCTATTG | TACCTCGCTGCCCTCCT | 124 |
| OPN | CCGTGGGAAGGACAGTT | TTGCTCTCATCATTGGCTT | 108 |
| PPARG | CAGGCCGAGAAGGAGAA | CTTTGGTCAGCGGGAAG | 133 |
| Smurf1 | CCACCAGACCAAAAGCAT | GGGAGCCACCAACAAAA | 144 |

Table 2. Antibodies

| Proteins | Cat.No. | Company |
| --- | --- | --- |
| LMCD1 | ab179454 | Abcam |
| Collagen I | ab34710 | Abcam |
| RUNX2 | #12556 | Cell signal technology |
| Sp7 | ab22552 | Abcam |
| Smad1/5 (phospho Ser463/465) | #9516 | Cell signal technology |
| Smad1/5 | #6944 | Cell signal technology |
| Ubiquitin | ab7780 | Abcam |
| Smurf1 | ab38866 | Abcam |
| β-actin | HRP-60008 | Proteintech |
| Secondary antibody | 111-035-003 | Jackson |
