# Supplementary material for: LMCD1 promotes osteogenic differentiation of human bone marrow stem cells by regulating BMP signaling
Source: Cell Death Dis. 2019 Sep 9;10(9):647. doi: 10.1038/s41419-019-1876-7 (PMC6733937; doi:10.1038/s41419-019-1876-7)
Supplement: Supplementary file 2 — LMCD1 figure legends for supplementary [file 41419_2019_1876_MOESM2_ESM.docx]

**Figure legends for supplementary:**

**Fig S1. a** Representative Masson staining of the transplants from sh-Control group and sh-LMCD1 group. Black arrow represents newly formed bone. Scale bar represents 100µm. **b** Quantitative measurement of bone formation. **c** Representative Masson staining of the transplants from Vector group and LMCD1 group. Black arrow represents newly formed bone. Scale bar represents 100µm. **d** Quantitative measurement of bone formation. **e,f** Representative images of OCN immunofluorescence of each group. Scale bar represents 100µm. **g,h** Quantitative measurement of OCN immunofluorescence images of each group. **i,j** Representative images of PPARG immunofluorescence of each group. Scale bar represents 100µm. **k,l** Quantitative measurement of PPARG immunofluorescence images of each group.

**Fig S2.** **a** Relative BRE Luciferase activity was measured in sh-Control and sh-Smurf1 group. **b** The expression of RUNX2, Smad1/5 and p-Smad1/5 (Ser463/465) were measured by western blotting in sh-Control and sh-Smurf1 group. **c** The expression of RUNX2, Smad1/5 and p-Smad1/5 (Ser463/465) were measured by western blotting with the depletion of LMCD1 or depletion of both LMCD1 and Smurf1.
