# Supplementary figures and images for: LMCD1 promotes osteogenic differentiation of human bone marrow stem cells by regulating BMP signaling
Source: Cell Death Dis. 2019 Sep 9;10(9):647. doi: 10.1038/s41419-019-1876-7 (PMC6733937; doi:10.1038/s41419-019-1876-7)

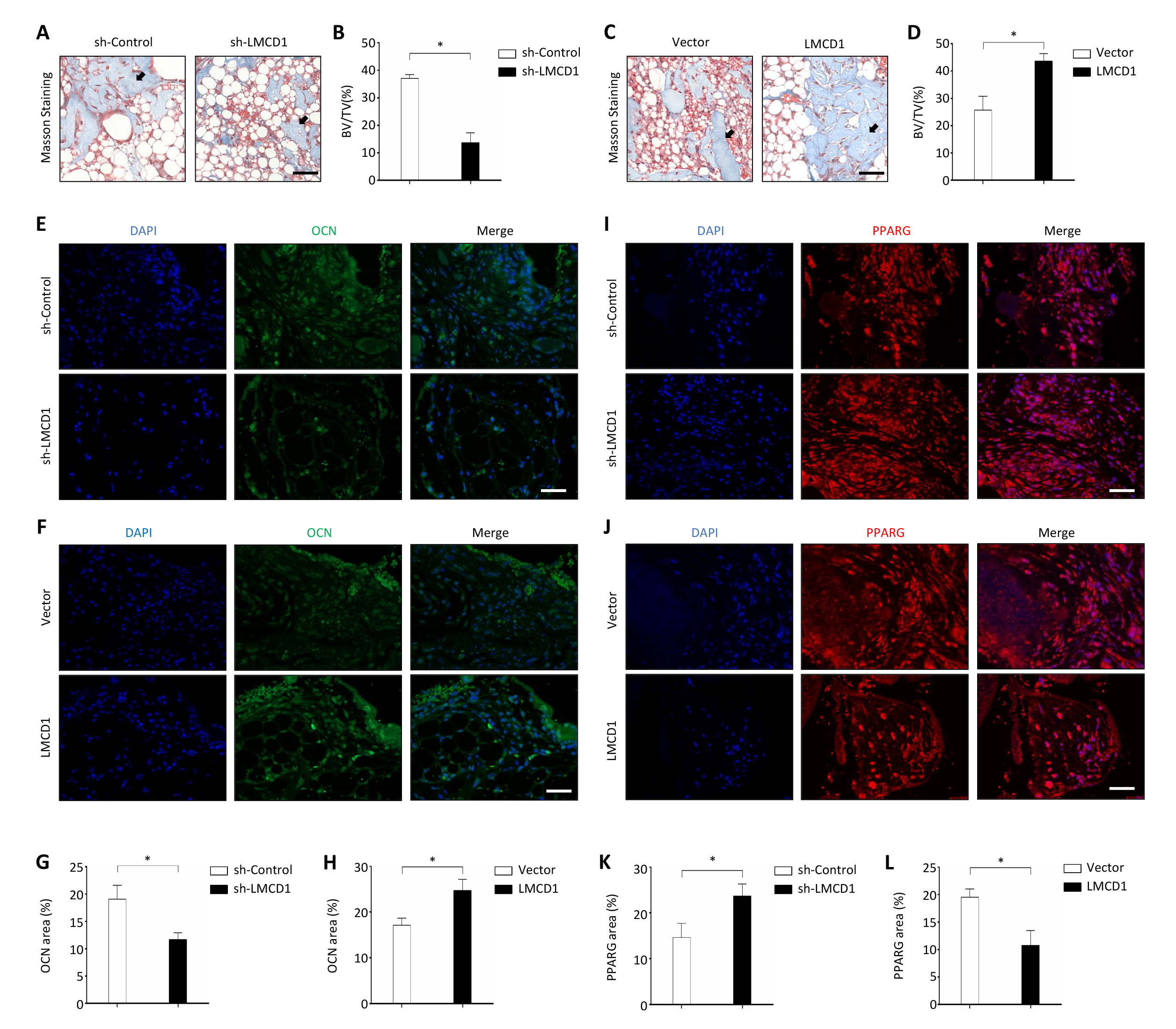

Supplement: Supplementary file 3 — Figure S1 [file 41419_2019_1876_MOESM3_ESM.tif]

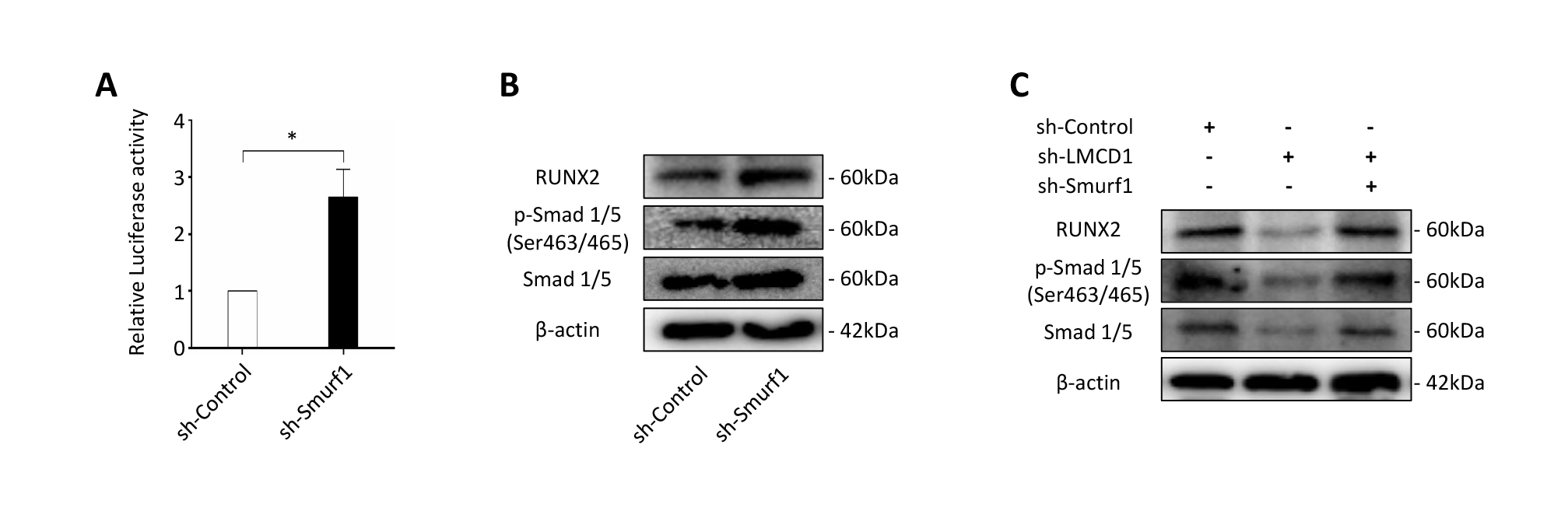

Supplement: Supplementary file 4 — Figure S2 [file 41419_2019_1876_MOESM4_ESM.tif]
